# Fibrose rétropéritonéale au CHU Ibn Sina de Rabat: à propos de 18 cas

**DOI:** 10.11604/pamj.2021.38.149.21666

**Published:** 2021-02-10

**Authors:** Abdoulazizi Bilgo, Moussokoro Hadja Koné, Youssef Lamzaf, Anissa Benjafaar, Amine Saouli, Tarik Karmouni, Khalid El Khader, Abdellatif Koutani, Ahmed Ibn Attya Andaloussi

**Affiliations:** 1 Service d´Urologie B, CHU Ibn Sina de Rabat, Rabat, Maroc,; 2 Faculté de Médecine et de Pharmacie, Université Mohammed V de Rabat, Rabat, Maroc,; 3 Service de Néphrologie et Transplantation, CHU Ibn Sina de Rabat, Rabat, Maroc

**Keywords:** Fibrose rétropéritonéale, insuffisance rénale obstructive, corticoïdes, Retroperitoneal fibrosis, obstructive renal failure, corticosteroids

## Abstract

La fibrose rétropéritonéale (FRP) est une pathologie rare caractérisée par la formation d´une plaque fibro-inflammatoire dans l´espace rétropéritonéale en avant de l´aorte abdominale, responsable d´un engainement des uretères. Le tableau clinique n´est pas spécifique et la pathologie est souvent révélée par une uropathie obstructive. Nous avons mené une étude descriptive rétrospective étendue sur 10 ans, de janvier 2006 à décembre 2016, réalisée dans les services d´urologie B et de néphrologie du Centre Hospitalier Universitaire Ibn Sina. Nous avons inclus 18 patients dans l´étude dont 11 hommes et 7 femmes, avec un âge moyen de 51,4 ans ± 11,2. Le diagnostic a été révélé par des douleurs lombaires chez 14 patients. L´insuffisance rénale obstructive était présente chez 15 de nos patients et le diagnostic de FRP a été posé par l´uroscanner. Le bilan étiologique notait des antécédents de néoplasies pour 2 patients, de pathologies inflammatoires pour 2 autres patients et une chirurgie rétropéritonéale chez un patient. On notait l´association de prises médicamenteuses chroniques chez plus de la moitié des patients. Le traitement comportait un double volet: une sonde double J systématique chez tous les patients et un traitement systémique par de la corticothérapie et des immunosuppresseurs selon le profil évolutif. Le traitement par urétérolyse a été réalisé d´emblée chez 3 patients. L´évolution a été favorable avec une nette amélioration de la fonction rénale chez 12 patients. La rechute est survenue chez 2 patients après 2 ans de suivi. L´association de signes généraux à une insuffisance rénale obstructive doit faire évoquer le diagnostic de FRP. La recherche de causes secondaires devrait être systématique, avec un accent particulier à mettre sur le syndrome d´hyper IgG4 et les pathologies néoplasiques.

## Abstract (en)

Retroperitoneal fibrosis (RPF) is a rare disease characterized by the formation of a fibro-inflammatory plaque in the retroperitoneal space in front of the abdominal aorta. It is responsible for the sheathing of the ureters. It is characterized by unspecific clinical signs, and it is often detected based on obstructive uropathy. We conducted a retrospective, descriptive study in the Department of Urology B and in the Department of Nephrology of the Ibn Sina University Hospital over a period of 10 years from January 2006 to December 2016. The study enrolled 18 patients, including 11 men and 7 women, with an average age of 51.4 years ± 11.2. Diagnosis was based on lumbar pain in 14 patients. Obstructive renal failure was reported in 15 patients and the diagnosis of RPF was based on uroscanner. Etiological assessment revealed 2 cases with a history of neoplasia, 2 cases of inflammatory disease and 1 case of retroperitoneal surgery; chronic drug intake was noted in more than half of the patients. In all patients, treatment was based on double J stent placement, while systemic treatment with corticosteroids and immunosuppressants was used on the basis of the evolutionary profile. Ureterolysis was immediately performed in 3 patients. Outcome was favorable, with significant improvement in renal function in 12 patients. A relapse occurred in 2 patients after 2 years of follow-up. Retroperitoneal fibrosis (RPF) should be suspected in patients with general signs associated with obstructive renal disease. Secondary causes should be systematically investigated, with particular emphasis on hyper IgG4 disease and neoplastic diseases.

## Introduction

La fibrose rétropéritonéale (FRP) est une pathologie rare caractérisée par la formation d´une plaque fibro-inflammatoire dans l´espace rétropéritonéale en avant de l´aorte abdominale, responsable d´un engainement des uretères. Le tableau clinique n´est pas spécifique et la pathologie est souvent révélée par une uropathie obstructive. Elle est primitive dans 2/3 des cas et secondaire dans le tiers restant [1]. Le scanner et l´imagerie par résonance magnétique (IRM) constituent les examens clés pour l´orientation diagnostique. Le traitement ne fait pas l´objet de consensus néanmoins on s´accorde sur l´usage de la corticothérapie, des immunosuppresseurs et/ou de l´hormonothérapie en association avec les moyens chirurgicaux allant des techniques de drainage des voies urinaires supérieures à l´urétérolyse [2]. L´objectif de notre travail est de décrire les modalités diagnostiques et thérapeutiques de la FRP au Centre Hospitalier Universitaire Ibn Sina (CHUIS) de Rabat.

## Méthodes

Nous avons mené une étude descriptive rétrospective étendue sur 10 ans, de janvier 2006 à décembre 2016, réalisée dans les services d´urologie B et de néphrologie du centre hospitalier universitaire Ibn Sina (CHUIS). Les données ont été recueillies à partir des dossiers médicaux des patients. Nous avons inclus dans l´étude tous les patients ayant une FRP sans distinction de l´étiologie. Les dossiers médicaux incomplets ont été d´emblée exclus de l´étude. Le diagnostic de FRP a été retenu devant la présence à l´imagerie d´un manchon fibreux rétropéritonéal périvasculaire associé ou non à la présence d´une insuffisance rénale et/ou d´un syndrome inflammatoire biologique. Après l´introduction du traitement adapté, le suivi des patients était mensuel sur une durée de 6 mois puis ensuite semestrielle avec un bilan biologique et un scanner de contrôle. Les variables recueillies ont été analysées grâce aux tests statistiques adaptées sur SPSS 20.

## Résultats

Les données cliniques et thérapeutiques sont regroupées dans le Tableau 1. Nous avons inclus dans l´étude 18 dossiers de FRP dont 11 hommes et 7 femmes, pris en charge au sein des 2 services. Les patients inclus dans l´étude avaient un âge moyen de 51,4 ans ± 11,24. Le motif de consultation le plus fréquent était les douleurs abdominales ou lombaires, retrouvées chez 14 patients. Les signes généraux comme la fièvre et l´amaigrissement étaient retrouvés chez 3 des patients et la symptomatologie locorégionale (varicocèle, hydrocèle, œdème des membres inférieurs et thrombose veineuse profonde) chez 7 patients. Le bilan biologique initial retrouvait constamment un syndrome inflammatoire avec une ascension de la vitesse de sédimentation (VS) et de la *C reactive protein* (CRP). Une insuffisance rénale aiguë a été retrouvée chez 15 patients avec un taux de créatinine médian de 68 mg/l (28,9-121). L´insuffisance rénale et les lombalgies donnaient lieu systématiquement à la réalisation d´une échographie. Elle a été réalisée chez 16 patients, et elle a révélé une urétéro-hydronéphrose chez 14 patients. La totalité des patients disposait d´un scanner qui révélait à fois la plaque de fibrose et l´urétéro-hydronéphrose (UHN) chez 16 patients et retrouvait uniquement de la fibrose sans dilatation des voies excrétrices rénales chez 2 patients. Sur le plan étiologique, les formes idiopathiques représentaient 78% de la série soit 14 patients, et 4 cas de fibrose étaient classées secondaires au vu des antécédents de spondylarthrite ankylosante (SPA), de chirurgie pour hernie discale, de lymphome non-Hodgkinien et de néoplasie du col utérin avec envahissement du rétropéritoine. Néanmoins, 8 patients avaient des antécédents de prise médicamenteuse chronique (3 patients sous anti-inflammatoires non stéroïdiens, 2 sous antidiabétiques oraux, 1 patient sous inhibiteur calcique et un autre sous L-dopa pour maladie de Parkinson). Il n´a pas été retrouvé d´anévrysme de l´aorte abdominale, mais 5 patients présentaient des calcifications aortiques athéromateuses. Deux patients ont eu un dosage des anticorps anti-DNA, anti-nucléaires, et anti-cytoplasme des polynucléaires dont les titres étaient positifs mais faibles. Un patient a bénéficié du dosage des IgG4 sériques dont le taux est revenu normal.

**Tableau 1:** caractéristiques cliniques, paracliniques des patients et options thérapeutiques

Paramètres recueillis	Données
**Age (années)**	51,4±11,2
**Sexe (H/F)**	11/7
Signes cliniques (n=18)*	
Douleurs lombaires	14
Amaigrissement	3
Fièvre	3
TVP	1
OMI	2
Varicocèle	2
Hydrocèle	2
Oligo-anurie	2
**Découverte fortuite (n=18)**	2
**Comorbidités (n=18)**	
HTA	3
Tabac	4
Diabète	1
Athérome connu	1
**ATCD (n=18)**	
Médicamenteux	8
Radiothérapie/Chimiothérapie	2
Chirurgie retro péritonéale	1
Pathologies rhumatismales associées	2
**Echographie (n=16)**	
UHN unilatérale	7
UHN bilatérale	7
Pas d'UHN	2
**TDM (n=18)**	
UHN+ FRP	16
FRP sans UHN	2
Polyadénopathie	1
Anévrysme	0
Athéromes	5
**Modalités thérapeutiques des patients (n=18)**	
Sondes JJ	18
Néphrostomies	1
Corticoïdes seuls	12
Corticoïdes + Tamoxifène Corticoïdes + Immunosuppresseurs	1 1
Urétérolyse	3

(*un patient peut cumuler plusieurs symptômes, antécédents ou facteurs de risques. Chaque variable est étudiée sur l'ensemble des 18 patients de la série.)

**TVP**: thrombose veineuse profonde, **OMI**: œdème des membres inférieurs, **HTA**: Hypertension Artérielle, **ATCD**: antécédents, **UHN**: urétéro-Hydronéphrose, **TDM**: tomodensitométrie.

La biopsie de la plaque a été réalisée chez 9 patients dont 6 par guidage radiologique (scanner) et 3 patients au cours d´une urétérolyse. L´aspect histologique des biopsies revenait en faveur d´un tissu fibreux ponctué de cellules inflammatoires polymorphes fait d´éléments lympho-histiocytaires et de polynucléaires neutrophiles, sans caractère spécifique. Elle n´orientait pas la prise en charge. La prise en charge initiale consistait au drainage urgent des cavités rénales. Après drainage, 14 patients ont été mis sous corticothérapie seule par Prednisone à la dose initiale de 60mg/j pendant 1 à 2 mois, puis une dégression progressive par pallier de 10mg chaque mois jusqu´à une dose journalière de 10-5mg/jour. La durée moyenne du traitement était de 18 mois. Au bout de 2 mois de corticothérapie, l´évolution a été marquée par une nette régression du syndrome inflammatoire chez 10 des 14 patients. Douze patients avaient une amélioration de la fonction rénale de plus 50% par rapport aux valeurs initiales, tandis que 2 patients présentaient des taux de créatinine stationnaires et 3 ont nécessité une séance de dialyse. Trois patients ont bénéficié d´une urétérolyse en première intention. Une rechute a été notée chez 2 patients au bout 2 ans conduisant à une association corticothérapie-tamoxifène et corticothérapie-immunosuppresseurs avec changement itératif des sondes double J. Aucun décès directement imputé à la FRP ou à son traitement n´a été enregistré. Deux patients ont développé respectivement un diabète cortico-induit et une hypertension artérielle au cours du traitement.

## Discussion

La FRP est une pathologie rare dont l´incidence est estimée à 0,4 à 1/200 000 personnes [3], décrite pour la première fois par Albarran avant d´être établie comme entité clinique suite à la publication d´Ormond qui lui donne son nom. C´est une pathologie prédominant chez l´homme de 40 à 60 ans. Dans notre étude, la moyenne d´âge est de 51,4 ans ± 11,24. La présentation clinique habituelle est celle de vagues douleurs abdominales avec discrète altération de l´état général évoluant depuis plusieurs mois. Les signes urologiques spécifiques restent fréquents, expliqués par la compression des organes de voisinage par la fibrose. On observe ainsi des coliques néphrétiques, une hydrocèle, une claudication intermittente des membres inférieurs, des thromboses veineuses profondes des membres inférieurs [4]. Dans notre série, 7 de nos patients présentaient des signes de compression. Ces signes cliniques de compression s´expliquent par la transformation progressive de la graisse de la région péritonéale en une chape fibreuse qui engaine les organes avoisinants.

## La pathogenèse reste cependant obscure, plusieurs considérations ont été apportées au fil du temps. Ainsi, Parums et Mitchinson l´expliquait par une réaction inflammatoire locale mal contrôlée au cours de l´athérosclérose aortique [5]. Cependant, tous les patients ayant une FRP n´ont pas d´athérosclérose aortique comme 13 des patients de notre série. De plus, la présence de signes généraux chez plus de la moitié des patients ne se satisfait pas de la théorie de l´inflammation locale exagérée. La théorie de la manifestation locale d´une pathologie systémique auto-immune est donc évoquée. Etant donné que la FRP s´accompagne parfois d´affections vasculaires touchant l´aorte thoracique ou les artères mésentériques, cette théorie reste possible. La présence d´une pathologique systémique est d´autant plus plausible que des signes généraux sont présents et que la FRP est quelques fois accompagnée d´autre maladies auto-immunes [5]. Nous avons dans notre série deux patients porteurs de spondylarthrite ankylosante, cependant la relation de cause à effet est difficile à établir

Les pathologies néoplasiques peuvent aussi être à l´origine d´une FRP. Les néoplasies primitifs de la région rétro péritonéale sont à l´origine FRP comme chez notre patient qui avait un lymphome de Hodgkin ou celle qui avait un néoplasie du col utérin. Il peut également s´agir d´une FRP due à une réaction paranéoplasique induite par les tumeurs carcinoïdes [5]. Une autre cause récemment décrite de la FRP est représentée par le syndrome d´hyper-IgG4. Cette entité de description récente est une atteinte fibro-inflammatoire de différents organes, associée le plus souvent à une élévation des IgG4 sériques [6]. Le diagnostic repose non seulement sur le dosage des taux sériques d´IgG 4 mais aussi sur les résultats histopathologique et immunohistochimique de la biopsie [4]. Un patient de la série a eu un dosage sérique d´IgG4 revenu normal.

Les FRP secondaires ne représentent cependant que 30% des FRP. La majorité des FRP diagnostiquées sont idiopathiques. Il peut être retrouvé des prises médicamenteuses notamment la L-dopa, les bétabloquants qui pourraient être des facteurs favorisants de l´apparition d´une FRP [7]. Parmi nos patients qui avaient une FRP idiopathique, 8 patients c´est à dire plus de 50% avaient des prises médicamenteuses chroniques, traduisant la fréquence de l´association entre prises médicamenteuses et FRP. Sur le plan thérapeutique, la dérivation des urines a été la première mesure prise pour chaque patient qui avait une dilatation pyélocalicielle. Ils ont bénéficié de montées de sonde double J, permettant de soulager les reins.

Par la suite, un traitement à visée systémique a été entrepris. La corticothérapie est le traitement le plus utilisée mais il n´y a pas de consensus concernant le protocole thérapeutique. Jakhlal *et al*. décrivent une partie des protocoles de corticothérapie dans leur revue de la littérature [4]. Dans notre série, le protocole de corticothérapie était basé sur l´usage de la Prednisone à 1 mg/kg/jour pendant 1 à 2 mois selon la réponse initiale. Puis une dégression progressive jusqu´à l´arrêt. La durée totale du traitement était de 18 mois en moyenne avec une évolution rapidement favorable. Cependant les corticoïdes sont responsables d´effets secondaires importants d´où l´utilisation d´autres molécules. Ces stratégies d´épargne cortisonique font appel à l´Azathioprine, au Mycophenolate Mofetil ou au Tamoxifène [8]. Les résultats sont comparables par rapport à l´usage de la corticothérapie seule. Sur le plan chirurgical, l´urétérolyse constitue l´ultime recours devant les plaques persistantes après une thérapeutique médicale combinée à base de corticoïde et d´immunosuppresseur ou une monothérapie de longue durée, à raison de leur caractère plus sclérotique qu´inflammatoire [9]. Chez nos 3 patients, l´abord a été une laparotomie médiane avec intra péritonisation des uretères, sans tentative d´ablation de la plaque de fibrose qui est une attitude à proscrire.

## Conclusion

La fibrose rétropéritonéale reste un diagnostic radiologique, avec l´insuffisance rénale aiguë obstructive comme principale urgence chez les patients. La biopsie n´est pas obligatoire avant l´initiation d´un traitement médical vu les résultats obtenus dans notre série et la revue de la littérature. Mais les formes secondaires ne doivent pas être méconnues au vu des pathologies à IgG4 souvent associées. La corticothérapie reste le gold standard du traitement, associée au drainage. Le dosage d´IgG4 et le bilan inflammatoire garde une place pour la surveillance sous traitement. Les rechutes restent fréquentes et justifient un suivi au long court avec éventuellement l´urétérolyse comme solution de dernier recours.

### Etat des connaissances sur le sujet

- La fibrose rétropéritonéale est une pathologie fibro-inflammatoire chronique dont le traitement repose sur la corticothérapie et le drainage urinaire;
- L´attitude commune étant de réaliser des biopsies avant le démarrage du traitement.

### Contribution de notre étude à la connaissance

- Il n´y a pas d´intérêt à faire des biopsies systématiques avant le traitement;
- Il faut la recherche d´un contexte de maladie fibrosclérosante à IgG4 au moment du diagnostic.
